# Characteristics of Elliptical Vibration-Assisted Cutting with Variations in Tilt Angle of Elliptical Locus

**DOI:** 10.3390/mi14071426

**Published:** 2023-07-15

**Authors:** Senbin Xia, Ziqiang Yin, Cheng Huang, Yawen Guo, Chao Zhang

**Affiliations:** 1 State Key Laboratory of Precision Electronic Manufacturing Technology and Equipment, Guangdong University of Technology, Guangzhou 510006, China; xiasenbin@163.com (S.X.);; 2 School of Electromechanical Engineering, Guangdong University of Technology, Guangzhou 510006, China

**Keywords:** elliptical vibration-assisted cutting, tilt angle, elliptical locus, diamond cutting

## Abstract

Elliptical vibration-assisted cutting (EVAC), one of the advanced micromachining methods, enables results not possible with traditional ultra-precision machining. It is considered to be one of the most viable options for manufacturing micro/nanostructured surfaces. However, it is difficult to control the elliptical locus with different tilt angles; therefore, previous studies have primarily focused on fixed locus and investigated the effects of the amplitude and frequency on machining performance. In addition, tilt angle is an important factor affecting the characteristics of EVAC. To maximize the cutting performance of EVAC, the cutting characteristics of EVAC with variations in tilt angle of elliptical locus are investigated. The mathematical model of elliptical trajectory based on different tilt angles is established via geometric analysis. The effects of the different tilt angle (0–180°) on cutting forces, chip formation, defect generation and surface roughness are observed and theoretically analyzed in microgroove experiments. The experimental results show that the tilt angle has a significant effect on the cutting force, chip formation, defects and surface roughness. The best cutting performance can be obtained at the tilt angle of 30°, while the worst is recorded at 90°. The results can provide a valuable reference for further comprehensive studies to maximize the cutting performance of EVAC.

## 1. Introduction

Micro/nanostructured surfaces can provide advanced and interesting functions. A large number of applications of these functional structured surfaces in different fields, including optics [1,2], solar energy technology [3], and measuring systems [4], provide a challenge for accurate and efficient processing. To overcome this problem, many machining methods have been demonstrated [5,6]. However, there are still various limitations, such as a low processing efficiency, large cutting force, and severe tool wear. Therefore, the development of more advanced manufacturing technology has been a research topic that has attracted much attention in recent years.

Elliptical vibration-assisted cutting (EVAC), one of the advanced micro-machining methods, enables results not possible with traditional ultra-precision machining. It is based on one-dimensional vibration-assisted machining (1D-VAM), vertically superimposing another dimensional vibration in the direction of cutting depth [7,8]. As a result, an elliptical tool path will be generated via the synthesis of these two high-frequency vibrations. Additionally, EVAC exhibits a better machining performance than that of the 1D-VAM process. Due to its excellent machining performance, an increasing number of scholars have devoted increasing amounts of attention to EVAC technology.

Farrus N et al. conducted a series of cutting trials involving the fabrication of micro V-grooves by means of multi-pass ultraprecise single point cutting to demonstrate the positive effect of EVC motions on cutting force reduction and stabilization [9]. Pan Y et al. studied the wear of single crystal diamond tool and its influence on surface roughness in elliptical vibration cutting [10]. Zhang C et al. and Kim et al. experimentally investigated the effects of the ultrasonic elliptical vibrations as compared to those of the ordinary cutting method. The results show that EVC improves the microgroove turning process with respect to cutting forces, microgroove surface roughness, and burr formation for difficult-to-cut materials [11,12]. Li L et al. investigated the vibration detection and provided technical solutions for industrial monitoring systems [13,14]. Nath C et al. investigated the effects of tool vibration frequency, tool vibration amplitude and workpiece cutting speed using the UVC method. Additionally, the authors highlighted that the tool-workpiece contact ratio (TWCR) plays a key role in the UVC process [15]. Celaya and Lopez de Lacalle et al. investigated the advantages and drawbacks of ultrasonic-assisted turning (UAT), with a focus on the effects of tool vibration on surface quality. The experimental results show that UAT can improve the surface quality; especially important, it can be improved up to 40% when the vibration is applied in the direction of the cutting speed [16]. Suarez A et al. investigated the effects of ultrasonic vibration-assisted milling on important aspects such as material surface integrity, tool wear, cutting forces and fatigue resistance [17].

Suzuki et al. reported a new ultraprecision sculpturing method that will generate the contours of the microstructure by varying the elliptical vibration amplitude, and successfully performed the nanosculpturing experiments on hardened steel [18]. Zhang J et al. investigated the machining performance for hardened steel at micro/nanoscales and the limitations in nanoscale machining utilizing elliptical vibration cutting with amplitude control sculpturing technology [19]. Zhou et al. proposed the double-frequency elliptical vibration cutting method for freeform surface diamond machining, which provides the high-frequency elliptical vibration to improve the machinability in material removal and the low-frequency reciprocating movements for the freeform surface profile generation [20,21]. Kim et al. investigated the chip and burr formation in micro V-grooves using EVC. The authors point out that the reversal of the direction of the frictional force and the marked increase in the shear angle are the two major characteristics of the EVC process [22]. Additionally, Kim G et al. also experimentally investigated the effect of the tilt angle of an elliptical tool trajectory on the cutting force [23].

In summary, the previous studies focus on investigating the machining characteristics of EVC performers at a fixed shape of the elliptical tool path, as well as on changing the elliptical vibration amplitude and frequency to implement ultraprecision sculpturing technology. However, the shape of the elliptical path is the most important factor affecting the characteristics of EVAC.

Therefore, this paper investigates the characteristics of EVAC with variations in the tilt angle of a elliptical locus. The shapes of the elliptical tool paths are controlled by changing the magnitude and phase of voltages supplied by the piezoelectric actuators. With the aluminum as the workpiece material, the mathematical model of the elliptical locus with different tilt angles is established, and the influence of the elliptical locus with different tilt angles on cutting force, chip formation, processing defects, and the roughness of the machining surface are investigated. This work, which focuses on the effect of tilt angle on cutting characteristics, can offer a helpful reference for the comprehensive study of EVAC and maximize the cutting performance of EVAC.

## 2. Elliptical Vibration-Assisted Cutting Device and Method

### 2.1. EVAC Device and Kinematical Analysis

**Figure 1:**
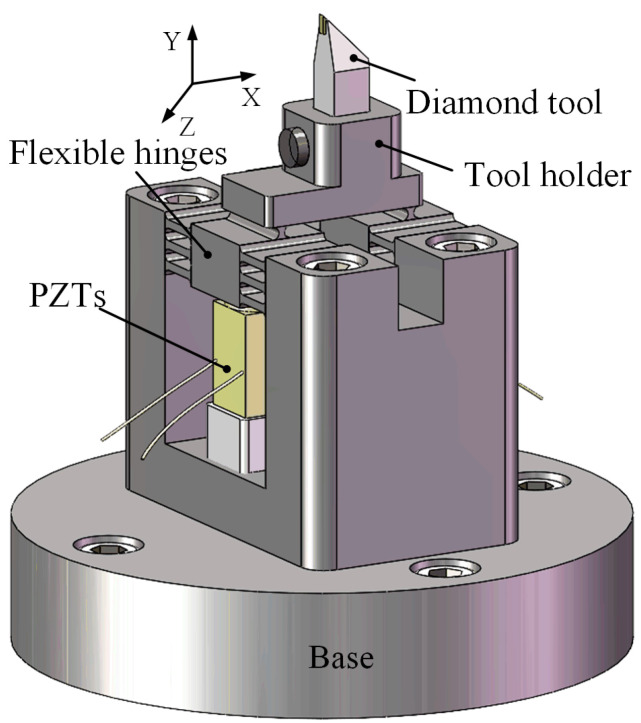
Elliptical vibration-assisted cutting device.

Although the resonant type elliptical vibration-assisted cutting (EVAC) enables researchers to work along an elliptical trajectory in resonant mode in order to obtain a high working frequency, it is difficult to precisely control/change the elliptical locus, and the working frequency is restricted to the resonant frequency. Compared to the resonant type, the non-resonant type EVAC can be easily controlled, allowing the elliptical locus of the cutting tool to be very accurate. Therefore, the non-resonant type EVAC device is designed to conduct a series of cutting experiments in this paper, as shown in Figure 1. An EVAC device comprises two parallel piezoelectric actuators, two flexible hinges, a cutting tool holder and a supporting base. The EVAC is made of Al7075 material and integrally formed via electric discharge wire cutting, which ensures a high accuracy of operation. Additionally, four countersunk holes are made on the supporting base to enable the installation of the holder. The flexible hinges are driven when the PZTs is applied the cosine voltage signals. As a result, the micro-displacement of the diamond tool tip is output.

**Figure 2:**
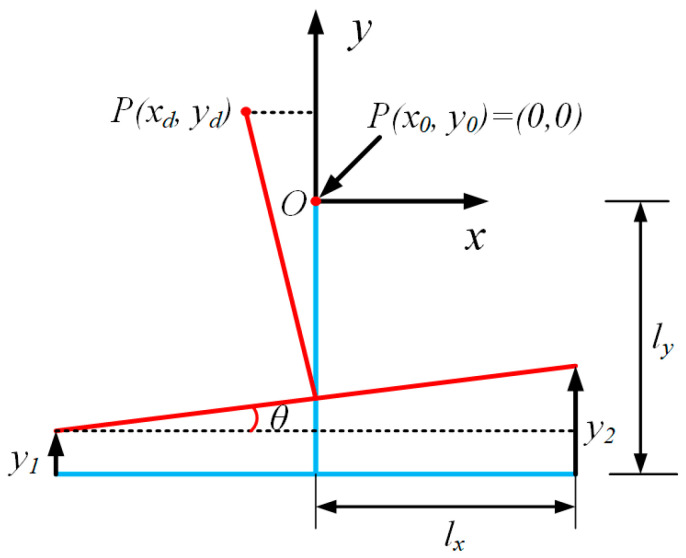
Kinematical schematic of EVAC.

Kinematic analysis of EVAC device is necessary for the subsequent control of elliptical trajectories. As shown in Figure 2, the T-shaped bar and tool holder are simplified as a rigid bar, and it is assumed that they undergo no deformation during the turning process.

In a global coordinate system *O*-*xy*, *P*(*x*_0_, *y*_0_) denotes the equilibrium position of the tool tip without any input displacement. *P*(*x_d_*, *y_d_*) represents the transient tool tip position when PZT-1 outputs a displacement $y_{1}$ and PZT-2 outputs a displacement $y_{2}$. According to the description of Figure 2, the following equations can be obtained:

(1) $$x_{d}=-l_{y}\sin\theta$$

(2) $$y_{d}=y_{1}+l_{x}\sin\theta +l_{y}\cos\theta -l_{y}$$

(3) $$\sin\theta =\left(y_{2}-y_{1}\right)/\left(2l_{x}\right)$$

where $l_{x}$ is half distance of the two PZTs in the *x* direction, and $l_{y}$ is the vertical distance between the PZT and the cutting tool edge in the *y* direction. θ represents the deflection angle between the PZT.

It should be noted that the PZTs are energized with the cosine voltages. Thus, the output displacements ($y_{1}$ and $y_{2}$) of the two PZTs can be calculated using Equations (4) and (5).

(4) $$y_{1}=A_{1}\cos\left(2\pi ft\right)$$

(5) $$y_{2}=A_{2}\cos\left(2\pi ft+\varphi \right)$$

where $A_{1}$ and $A_{2}$ are the vibration amplitudes along the y directions. f is the frequency, t represents the time, and φ is the phase shift.

In particular, the output displacements $y_{1}$ and $y_{2}$ are always kept at the microscale, which are significantly smaller than the dimensions of the T-shaped bar. Thus, the value of the θ can be assumed to be very small, as according to the small angle assumption, the cos⁡θ is approximately equal to 1. Substituting Equations (4) and (5) into Equations (1) and (2), Equations (1) and (2) can be simplified as Equations (6) and (7).

(6) $$x_{d}=\frac{l_{y}\left(A_{1}\cos\left(2\pi ft\right)-A_{2}\cos\left(2\pi ft+\varphi \right)\right)}{2l_{x}}$$

(7) $$y_{d}=\frac{A_{1}\cos\left(2\pi ft\right)+A_{2}\cos\left(2\pi ft+\varphi \right)}{2}$$

**Figure 3:**
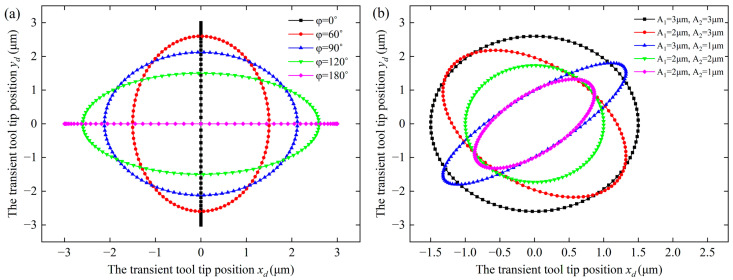
Simulation results of the elliptical cutting tool path: (**a**) $A_{1}=A_{2}=3\;µm$, with the different phases. (**b**) φ=60˚, with the variation in vibration amplitudes.

To verify and illustrate the generation of the elliptical cutting tool path, MATLAB is used to simulate the variation in the elliptical cutting tool path based on Equations (6) and (7). The frequency f is set to 1000 Hz, and $l_{x}$ and $l_{y}$ are assumed to be equal, while the phase and the vibration amplitudes are variable. As illustrated in Figure 3, the simulation results show that the elliptical cutting loci are successfully generated using Equations (6) and (7). Furthermore, the elliptical cutting locus are changed with the phase and the vibration amplitudes. This means that the elliptical cutting locus can be controlled by applying a suitable combination of the phase and the vibration amplitudes.

### 2.2. The Principle of the Elliptical Vibration-Assisted Cutting Process

**Figure 4:**
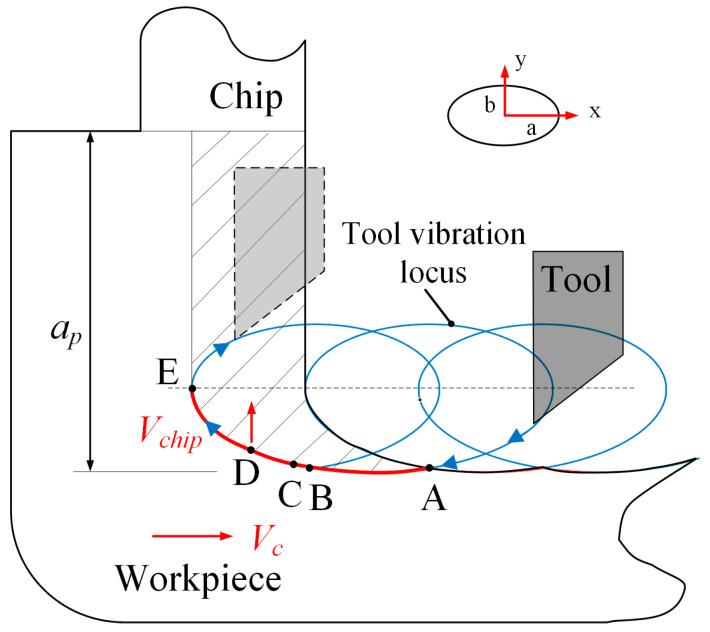
The principle of the EVAC process.

Figure 4 illustrates the schematic diagram of the EVAC process. The diamond tool is driven by applying the exciting voltage on the PZT actuators to generate the elliptical locus in the cutting plane. When coupled with a suitable cutting speed, a large number of overlapping elliptical loci will be formed.

In the EVAC process, the three most important features are the intermittent cutting, non-constant instantaneous cutting thickness, and the reversal of friction force between the tool and the chip [24]. To further illustrate these features, a single cutting cycle is analyzed. In a single cutting cycle, the diamond tool starts from point A, and ends at the ending point E along the cutting path (A-B-C-D-E). In particular, point B is the lowest point of the elliptical trajectory, which implies the maximum depth of the whole cutting process. It should be noted that the friction force between the tool and the chip will be reversed at point D. In this case, it will help the chips flow out during the D-E process. Finally, the tool separates from the workpiece at point E and starts the next cutting cycle. As a result, the intermittent cutting is generated.

The elliptical locus of the diamond tool can be obtained from Equations (4) and (5) above. Assuming that the workpiece cutting speed is $V_{c}$, then the motion of the diamond tool relative to the workpiece can be expressed as in Equation (8).

(8) $$\left\{\begin{array}{c} x\left(t\right)=a\cos(2\pi ft)-V_{c}t\\ y\left(t\right)=b\cos\left(2\pi ft+\varphi \right) \end{array}\right.$$

In addition, it should be noted that the speed ratio $R_{s}$ of the nominal cutting speed to the maximum tool vibration speed is an important parameter in the EVAC process. It can be written as in Equation (9). In particular, the intermittent cutting only occurs at the condition of $R_{s}<1$. To ensure the periodic separation between the tool and the workpiece, an $R_{s}<1$ should be maintained in the whole experiment.

(9) $$R_{s}=\frac{V_{c}}{2\pi fa}$$

### 2.3. Generation of the Elliptical Locus with Variations in the Tilt Angle

**Figure 5:**
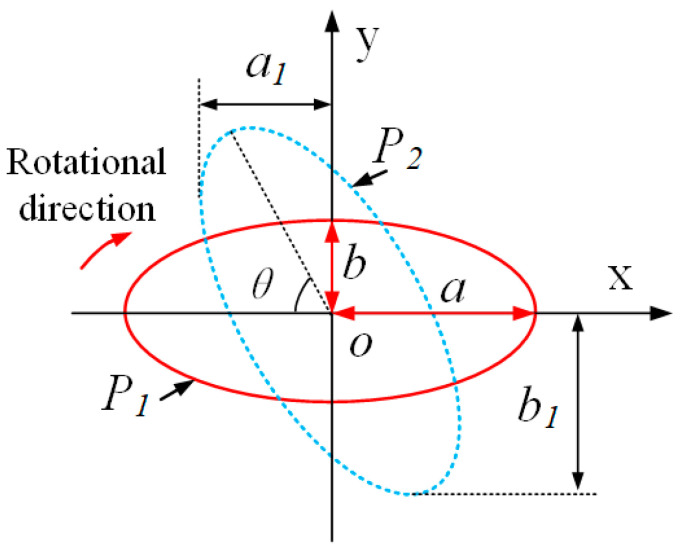
The geometrical relation of the ellipse with variations tilt angle.

To investigate the influence of the tilt angle in elliptical trajectory, it is necessary to establish a mode of the elliptical locus with variations in the tilt angle. In this paper, a mathematical mode is established via geometric analysis, as shown in Figure 5. The initial elliptical locus (*P*_1_) can be calculated using Equation (8) at the condition $V_{c}=0$, as shown in the red path in Figure 5. The deflection elliptical locus (*P*_2_) is obtained by rotating clockwise *θ* from type *P*_1_. In the coordinate system (*O*-*xy*), it is assumed that the ellipse (*P*_1_) has the major (*a*) and minor (*b*) axes (*a* ≥ *b*). Therefore, the coordinate expression after rotation can be given following Equation (10) according to the geometric relationship.

(10) $$\left[\begin{array}{c} x\prime (t)\\ y\prime (t) \end{array}\right]=\left[\begin{array}{cc} \cos\theta & -\sin\theta \\ \sin\theta & \cos\theta \end{array}\right]\left[\begin{array}{c} x(t)\\ y(t) \end{array}\right]$$

By substituting Equation (8) ($V_{c}=0$) into Equation (10), the corresponding parameters including the phase shift $\varphi_{1}$, and the vibration amplitudes ($a_{1}$ and $b_{1}$) can be calculated as follows:

(11) $$a_{1}=\sqrt{a^{2}\cos^{2}\theta_{1}+b^{2}\sin^{2}\theta_{1}}$$

(12) $$b_{1}=\sqrt{a^{2}\sin^{2}\theta_{1}+b^{2}\cos^{2}\theta_{1}}$$

(13) $$\varphi_{1}=\mathrm{accos}\left(\frac{c\sin\theta_{2}}{a_{1}}\right)-\arcsin\left(\frac{c\cos\left(\theta_{2}\right)}{b_{1}}\right)+90^{{}^{\circ}}$$

where, if the tilt angle θ is larger than $90^{{}^{\circ}}$, the major and minor axes will be changed. Therefore, the mathematical mode above should be configured as a different coefficient according to the tilt angle θ∈[0−180°], which can be obtained as follows:

(14) $$\theta_{1}=\theta_{2}=\theta ,\;c=b,\;\mathrm{where}\;\theta \leq 90^{{}^{\circ}}$$

(15) $$\theta_{1}=180^{{}^{\circ}}-\theta ,\;\theta_{2}=\theta -90^{{}^{\circ}},c=a,where\;\theta >90^{{}^{\circ}}$$

**Figure 6:**
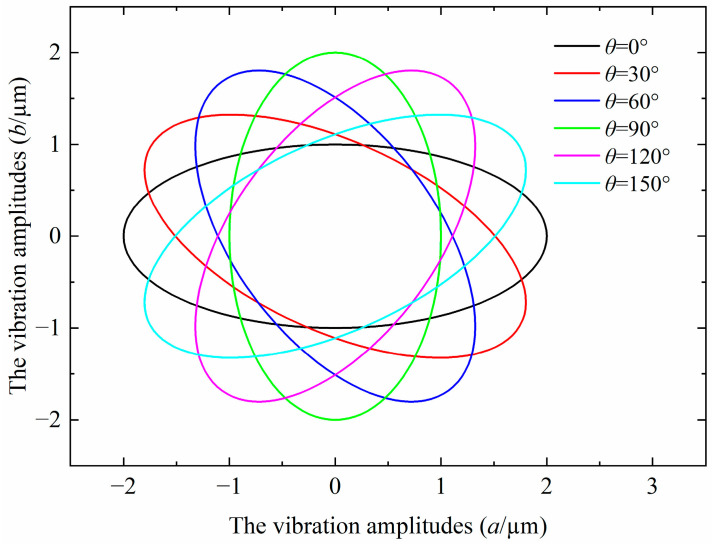
The elliptical locus with variations tilt angle (*a* = 2 µm, *b* = 1 µm).

Figure 6 illustrates the elliptical locus with variations in the tilt angle by applying Equations (11)–(15), in which the elliptical shape remains unchanged and rotates clockwise with the increase in the tilt angle. This implies that the mathematical mode of the elliptical locus with variations in the tilt angle agrees well with the theoretical results.

## 3. Experimental Setup

### 3.1. Elliptical Vibration-Assisted Cutting System Configuration

**Figure 7:**
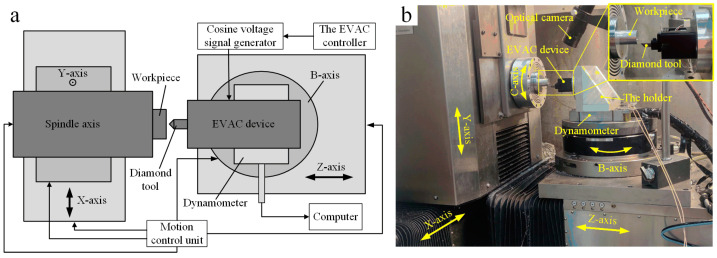
Elliptical vibration-assisted cutting system configuration: (**a**) the schematic block diagram, (**b**) the experimental setup.

The machining experiments are conducted on Moore Nanotech 350FG (Moore Nanotechnology Systems, Swanzey, NH, USA), as shown in Figure 7. It is an ultraprecision freeform generator with 5-axis. The machine utilizes three linear axes (*X*, *Y*, *Z*-axis) in conjunction with optional rotational axes (B-axis, C-axis). The *X*-axis, *Y*-axis, and *Z*-axis travels are 350 mm, 150 mm, and 300 mm, respectively. The optical system is applied to monitor the machining process.

The elliptical vibration-assisted cutting system consists of an elliptical vibration device and a signal generator with dual channels. In this paper, the signal generator (RIGOL DG4062, Rigol technologies, Suzhou, JS, CHN) with the output frequency of 1 Hz–60 MHz is selected to provide the excitation signals to the two PZTs simultaneously. Multicomponent dynamometer (KISTLER 9129AA, Kistler, Winterthur, CHE) is used to measure the cutting force. The diamond tool is mounted on the EVAC device, which will generate the elliptical trajectory driven by the EVAC device. In addition, the EVAC device is fixed on the dynamometer in order to record the cutting force.

### 3.2. Experiment Preparations

Aluminum alloy 6061 (Al6061, Shanghai Weilian Industrial, Shanghai, CHN) was selected as the experimental material due to its good cutting performance. Two manual crystal diamond tools (MCD, Sinjin Diamond, Seoul, KOR) are used to conduct the experimental studies. The first diamond tool has a tool radius *R* of 1.0 mm and a rake angle of 0°, while the cutting edge of the second diamond tool is flat. In order to evaluate the cutting performance of the elliptical locus with variations in the tilt angles, a single circular microgroove experiment was carried out under the same cutting conditions. Machining parameters and elliptical vibration parameters are shown in Table 1.

### 3.3. Performance Test of the Output Elliptical Locus

**Figure 8:**
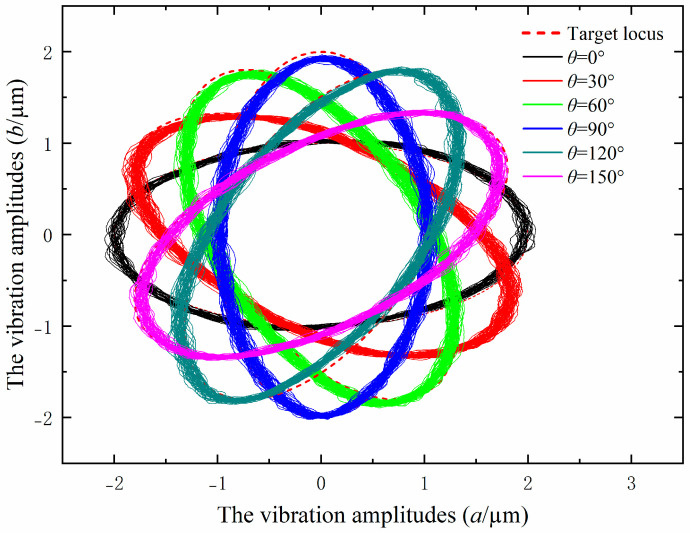
The output elliptical locus of the EVAC device.

To evaluate the effectiveness of the output elliptical locus of the EVAC device, the performance test was conducted before the machining experiments. The distances $l_{x}$ and $l_{y}$ are set as 11.5 cm and 42 cm in this paper. The displacements of the EVAC device are measured using the capacitive sensors (ACCUMEASURE D400, MTI Instruments, Albany, NY, USA) at the conditions shown Table 1. The measuring results are shown in Figure 8. It can be seen that the measured elliptic trajectories are highly consistent with the target locus, both in terms of the tilt angles and the vibration amplitudes. This implies that the mathematical model of the elliptical locus with variations in the tilt angle is efficient and can meet the experimental requirements.

## 4. Results and Discussion

### 4.1. Effects of the Tilt Angle on Cutting Force in Elliptical Vibration-Assisted Cutting

**Figure 9:**
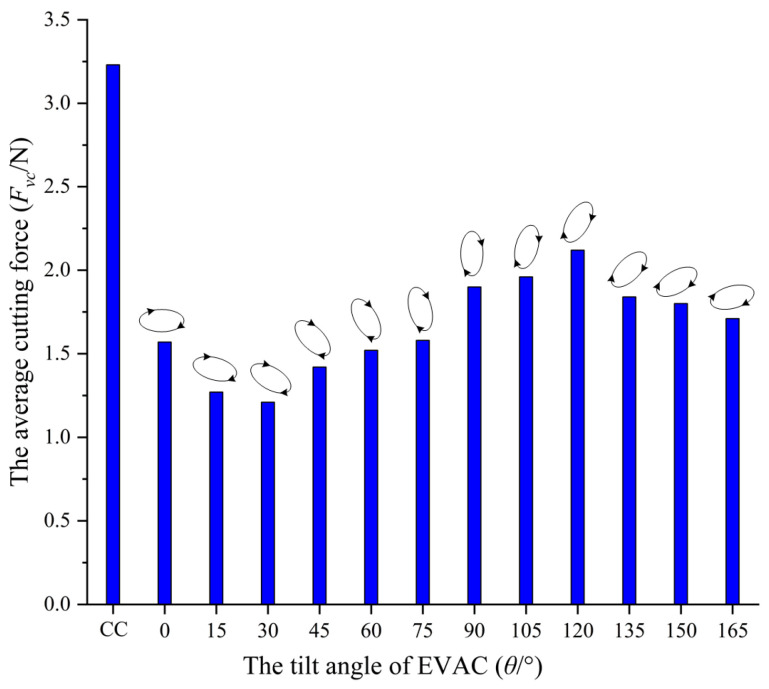
The cutting force variation with the tilt angle of EVAC.

In ultraprecision machining process, the cutting force is a factor that cannot be ignored. Smaller cutting forces have a significant impact on the improvement of diamond tool life and machined surface quality. To study the effects of the tilt angle of the EVAC on the cutting force, single-pass micro-grooves experiments are conducted using a diamond tool with the tool radius *R* of 1.0 mm and a rake angle of 0°, and the cutting conditions are shown in Table 1. The measuring results are shown in Figure 9.

From Figure 9, the cutting forces of the EVAC process are all significantly smaller compared to the conventional cutting process. There are two main reasons for this phenomenon. The first is that the EVAC process is a cyclical contact and separation process between the diamond tool and the workpiece, which results in much less contact time than that of conventional cutting. It implies that the average cutting force of EVAC will be reduced compared to that of conventional cutting. Secondly, the friction force between the diamond tool and the chip will be reversed (see point D in Figure 4) in the EVAC process, which is not the case in conventional cutting. This means that the cutting resistance between the tool and the chip in the EVAC process is less than that of conventional cutting.

**Figure 10:**
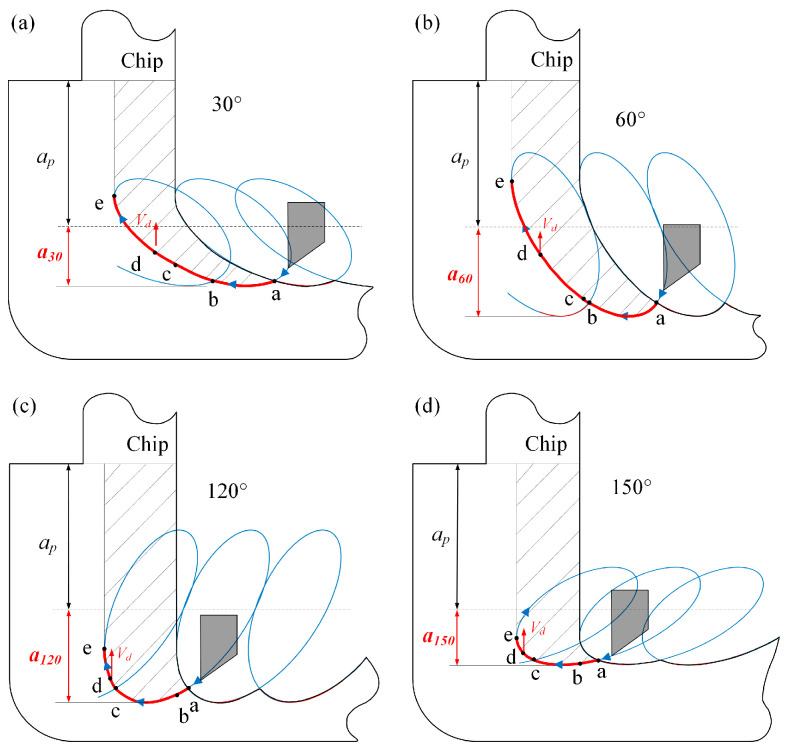
The cutting process varies with the tilt angle (*θ*) of EVAC: (**a**) *θ* = 30°, (**b**) *θ* = 60°, (**c**) *θ* = 120°, (**d**) *θ* = 150°.

Most importantly, the cutting force showed a high correlation with the tilt angle of EVAC. The cutting forces of the EVAC with the positive tilt angle (*θ* < 90°) are lower than that of the negative tilt angle (*θ* > 90°). In addition, the cutting forces are minimal at the tilt angle of 30°, while being maximal at the tilt angle of 120°. For further discussion, the EVAC process with different tilt angles was analyzed in depth, as shown in Figure 10.

As illustrated in Figure 10, there are two main factors that should be noted that influence the cutting forces. Firstly, the length of the cutting path (a–b–c–d–e) varies with the change in the tilt angle, especially in the D-E process. In the D-E process, the direction of the friction force between the tool and the chip is the same direction as that of the chip flow, which will help the chips flow out. This implies that the cutting force will decrease with an increase in the length of the D-E process. Secondly, the real depth of a cut consists of the nominal depth (*a_p_*) and the EVAC depth (*a_i_*). It can be noted that EVAC depth also varies with the change in the tilt angle. The real depth (*a_p_*+ *a_i_*) is minimal at the tilt angle of 30°, while being maximal at the tilt angle of 120°. This agrees with the change in the cutting force. In short, the cutting forces show a combined effect caused by the length of the cutting process and the real cutting depth.

### 4.2. Chip Formation with Different Tilt Angles

**Figure 11:**
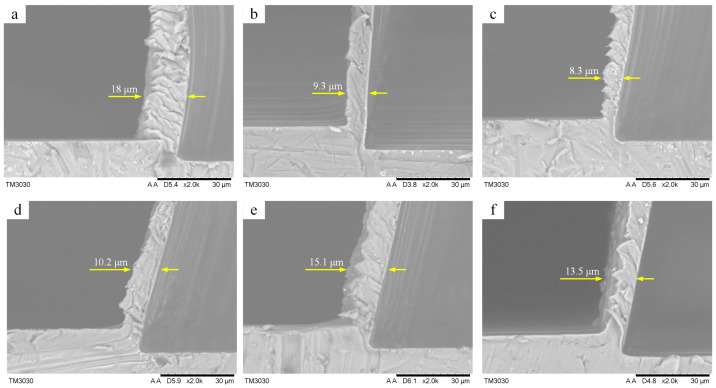
SEM images of chip formation at different tilt angles (*θ*): (**a**) conventional cutting, (**b**) *θ* = 30°, (**c**) *θ* = 60°, (**d**) *θ* = 90°, (**e**) *θ* = 120°, (**f**) *θ* = 150°.

Chip formation experiments were performed on the same workpiece with a flat diamond tool at different tilt angle (*θ*). The machining parameters are listed in Table 1. A scanning electron microscope (SEM) was used to measure the chip thickness, and some measure results are shown in Figure 11.

The experimental results shown that the chip thickness is less than that of the cutting depth (*a_p_* = 10 μm) under the positive tilt angle (*θ* < 90°), while larger than that at the negative tilt angle (*θ* ≥ 90°). Furthermore, the max chip thickness (15.1 μm) occurs in the tilt angle of 120° (see Figure 11e), while the min one (8.3 μm) appears at the tilt angle of 60° (see Figure 11c). The reason for these phenomena may be related to chip lifting effect. As shown in Figure 10 above, the tilt angle plays a key role in the chip lifting effect. When the tilt angle is less than 60°, the chip lifting effect increases along with the tilt angle. However, when the tilt angle is more than 90°, the chip lifting effect decreases with the tilt angle increase. As a result, the best chip lifting effect with the min chip thickness will be obtained at the tilt angle of 60°, and the worst chip lifting effect with the max chip thickness is found at the tilt angle of 120°.

### 4.3. Effects of the Tilt Angle on Machined Surface Defects

**Figure 12:**
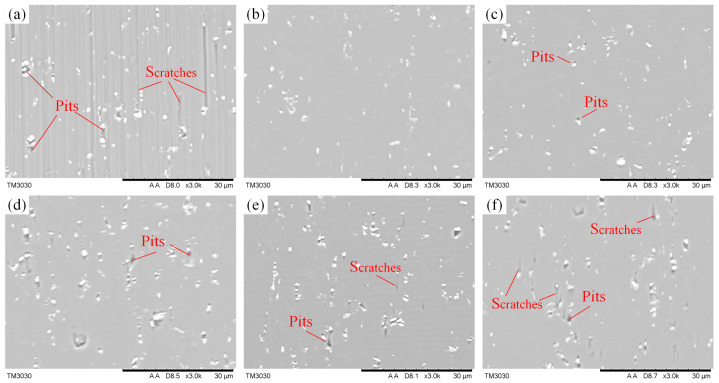
Defects on machined surfaces with different tilt angles (*θ*): (**a**) conventional cutting, (**b**) *θ* = 30°, (**c**) *θ* = 60°, (**d**) *θ* = 90°, (**e**) *θ* = 120°, (**f**) *θ* = 150°.

The machined surface of the microgrooves is illustrated in Figure 12. As can be seen from Figure 12a, the conventional cutting surface shows numerous surface defects such as long scratches and pits, whereas the machined surface of EVAC shows fewer defects, and the pits play a key role in these defects. In particularly, the number and size of defects on the machined surface varies with the tilt angle of EVAC, as shown in Figure 12b–f. The machined surfaces are very smooth and presents almost no defects when the tilt angle is less than 30°. However, the number and size of these pits increase along with the tilt angle (30° < *θ* ≤ 90°), while the scratches are almost nonexistent on the machined surfaces. When the tilt angle is larger than 90° (*θ* > 90°), a large number of pits remain on the machined surface can be observed, and the scratches slightly increase along with the increase in the tilt angle.

**Figure 13:**
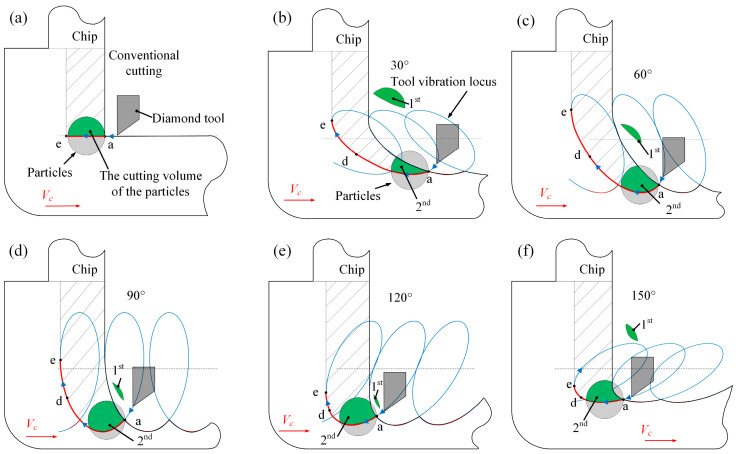
The mechanism of the particles’ influence on the machined surface with different tilt angles (*θ*): (**a**) conventional cutting, (**b**) *θ* = 30°, (**c**) *θ* = 60°, (**d**) *θ* = 90°, (**e**) *θ* = 120°, (**f**) *θ* = 150°.

These defects have been confirmed in our previous studies to be caused by the precipitate particles in aluminum alloy 6061 [25]. These particles are usually composed of alloying elements such as Fe, Mg_2_Si, SiO_2_ et al. In order to further analyze and discover the mechanism of the particles’ influence on the machined surface, the interaction mechanism between the diamond tool and the particle is studied. To ensure consistency, it is assumed in this paper that the middle position of the particle is the initial contact position between the diamond tool and the particle, as shown at point *a* in Figure 13.

From Figure 13, the cutting volume of the particles varies with the cutting method. In the conventional cutting process (see Figure 13a), the particle is not removed because of the large area of the cutting volume. As a result, the particle will be pushed forward by the diamond tool, which leaves long scratches on the machined surface. However, in the EVCA process with a small tilt angle (*θ* ≤ 30°), the particle will be divided into several small cutting areas (like in Figure 13b), which will allow the particles to be completely removed without leaving defects on the machined surface. When the tilt angle is between 30° and 90° (see Figure 13c,d), the cutting volume will increase along with the tilt angle. In this case, a small part of the particle will be cut off in the first cutting, while the larger part will be dug out of the workpiece in the second cutting so that the pits are generated on the machined surface. When the tilt angle is larger than 90° (*θ* > 90°), it is similar to the previous stage; the cutting volume is further increased, and thus the particle is scooped out, resulting in a large number of the pits on the machined surface. However, the elliptical locus is at a negative tilt. In this cutting process, the cutting path gradually became similar to the conventional cutting as the tilt angle increased (see Figure 13f), resulting in a higher cutting force of the diamond tool on the particle in the cutting direction. This is the reason for small scratches on the machined surface.

### 4.4. Effect of the Tilt Angle on Machined Surface Roughness

**Figure 14:**
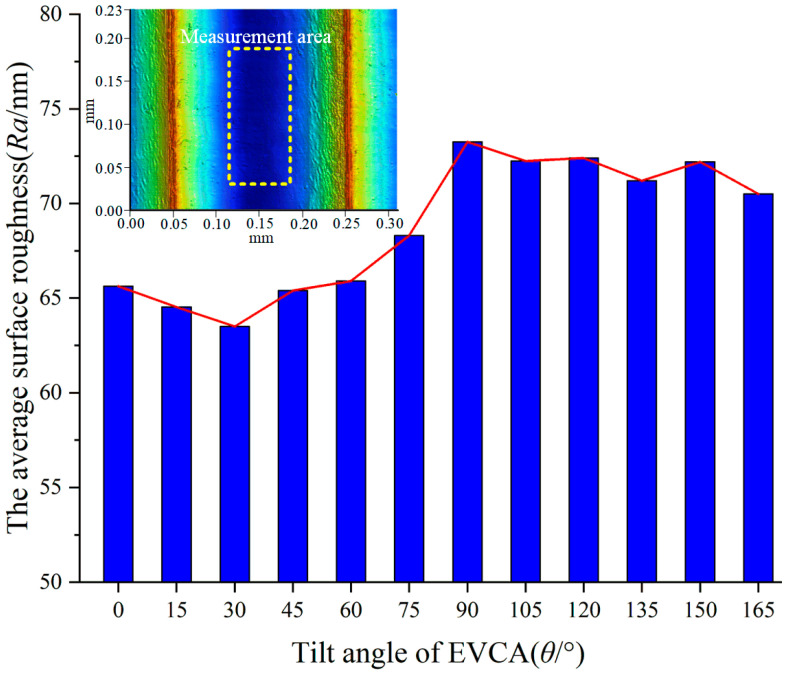
Surface roughness varies with tilt angle of EVCA.

The factors affecting the machined surface will eventually be reflected in the surface roughness. The parameters of surface roughness are traditionally divided into the average roughness *R_a_*, maximum roughness depth *R_max_* or maximum height of the profile *R_t_*, and the average maximum height *R_z_* [26]. To evaluate the cutting performance of elliptical vibration with different tilt angles, the average roughness *R_a_* of microgrooves was measured with an optical profiler (Bruker GT-X, Singapore Germany) in the same measurement area, and the average measuring results are shown in Figure 14. From Figure 14, the surface roughness decreases and then increases as the tilt angle increases (*θ* < 90°). When the tilt angle is larger than 90°, the surface roughness does not significantly change with the increase in the tilt angle. Furthermore, the surface roughness is minimized at a tilt angle of 30° and maximized at 90°.

This is mainly determined by the cutting characteristics with various tilt angles of the EVAC. From the above analysis, the machining process will be negatively impacted when the tilt angle is larger than 90°, which results in worse surface quality and higher surface roughness. However, if the tilt angle is selected below 60°, the lower cutting forces, smaller chip thickness, and fewer defects on the machined surface will be obtained. As a result, the lower surface roughness will be available. In particular, with the smallest cutting force and a smaller chip thickness, the best particle removal methods are responsible for the lowest surface roughness at the tilt angle of 30°. In addition, the defects (scratches and pits) play a key role in the surface roughness at the tilt angle of 90°.

## 5. Conclusions

In this paper, the cutting performance of the EVAC with different tilt angles is comprehensively investigated. By providing the cosine voltage through dual PZTs to output the displacement of the elliptical vibration device, the elliptical trajectory is generated. The mathematical model of EVAC with variations in the tilt angles was established based on theoretical analysis. A series of microgroove experiments were performed with variations in the tilt angle on Al6061 material. The effects of different tilt angles on cutting forces, chip formation, surface defects and surface roughness are analyzed. The important conclusions can be summarized as follows:

(1) To investigate the influence of the tilt angle on the elliptical trajectory, a mathematical model of the elliptical locus with variations in the tilt angle is analyzed and established via geometric analysis. A performance test of the output elliptical trajectory in the EVAC device is conducted. The test results show that the output elliptical trajectories are highly consistent with the target locus, both in terms of the tilt angles and the vibration amplitudes.
(2) The effects of different tilt angles on the cutting force are studied. The experimental results show that the cutting force presented a high correlation with the tilt angle of the EVAC. The cutting forces of the EVAC with the positive tilt angle (*θ* < 90°) are lower than those of the negative tilt angle (*θ* > 90°). In addition, the cutting forces are minimal at a tilt angle of 30°, while being maximal at a tilt angle of 120°. Through theoretical analysis, the reason found for these phenomena is that different tilt angles lead to different lengths of the cutting process and of the real cutting depth.
(3) The chip formation experiment results showed that EVAC generates a smaller chip thickness compared to that obtained through conventional cutting. Furthermore, when the tilt angle is less than 60°, the chip lifting effect increases along with the tilt angle. However, when the tilt angle is more than 90°, the chip lifting effect decreases with the tilt angle increase. As a result, the best chip lifting effect with the min chip thickness is determined at the tilt angle of 60°.
(4) Different tilt angles show significantly different effects on surface defects and surface roughness. The tilt angle with the lowest surface roughness and almost no defects is found (*θ* = 30°). Above the determined tilt angle (especially in *θ* = 90°), the machined surface will generate a lager number and size of scratches and pits, resulting in higher surface roughness. This implies that a suitable tilt angle will suppress the defects and achieve the best surface roughness.

## Figures and Tables

**Table 1:** Experimental parameters of the microgrooves.

Parameters	Value
Amplitude in cutting direction (*a*/µm)	2
Amplitude in cutting depth direction (*b*/µm)	1
Frequency (*f*/Hz)	1200
Tilt angle (*θ*/°)	0,15,30,45,60,75,90, 105,120,135,150,165
Cutting speed *V_c_* (mm/s)	2
Depth of cut *a_p_* (µm)	10

## Data Availability

All data needed to evaluate the conclusions in the paper are provided in the paper. Additional data related to this paper may be requested from the authors.
